# RBO Aleph: leveraging novel information sources for protein structure prediction

**DOI:** 10.1093/nar/gkv357

**Published:** 2015-04-20

**Authors:** Mahmoud Mabrouk, Ines Putz, Tim Werner, Michael Schneider, Moritz Neeb, Philipp Bartels, Oliver Brock

**Affiliations:** Robotics and Biology Laboratory, Department of Electrical Engineering and Computer Science, Technische Universität Berlin, Marchstraße 23, 10587 Berlin, Germany

## Abstract

RBO Aleph is a novel protein structure prediction web server for template-based modeling, protein contact prediction and *ab initio* structure prediction. The server has a strong emphasis on modeling difficult protein targets for which templates cannot be detected. RBO Aleph's unique features are (i) the use of combined evolutionary and physicochemical information to perform residue–residue contact prediction and (ii) leveraging this contact information effectively in conformational space search. RBO Aleph emerged as one of the leading approaches to *ab initio* protein structure prediction and contact prediction during the most recent Critical Assessment of Protein Structure Prediction experiment (CASP11, 2014). In addition to RBO Aleph's main focus on *ab initio* modeling, the server also provides state-of-the-art template-based modeling services. Based on template availability, RBO Aleph switches automatically between template-based modeling and *ab initio* prediction based on the target protein sequence, facilitating use especially for non-expert users. The RBO Aleph web server offers a range of tools for visualization and data analysis, such as the visualization of predicted models, predicted contacts and the estimated prediction error along the model's backbone. The server is accessible at http://compbio.robotics.tu-berlin.de/rbo_aleph/.

## INTRODUCTION

RBO Aleph is a novel web service for fully automated protein structure prediction, targeted at non-expert users. It provides services for template-based modeling, protein contact prediction and *ab initio* protein structure prediction.

RBO Aleph has a methodological emphasis on modeling difficult *ab initio* targets for which templates are not available. It therefore complements existing services that mainly focus on template-based modeling (1–6). The server automatically determines the most appropriate modeling method—template-based modeling or *ab initio* modeling—for a given protein and does not require the user to know about the availability of template structures in advance.

The effectiveness of RBO Aleph in modeling difficult targets stems from two key algorithmic features: first, our server uses residue–residue contacts to inform structure prediction. It begins by predicting residue–residue pairs in spatial proximity, also termed ‘contacts’. Such contact information can be extracted from the co-evolution signal of sequence homologs as well as from the physicochemistry of energy potentials (7). The synergistic combination of these two sources of information to predict contacts constitutes the first algorithmic feature. Second, RBO Aleph exploits the predicted contacts to guide the *ab initio* prediction. RBO Aleph uses an effective strategy to guide the conformational search toward regions likely to contain native-like structures. To achieve this, our method iteratively builds an approximate model of the energy landscape and uses it to guide sampling toward low energy regions (8). The contact information effectively smoothens the energy landscape, rendering conformational space search more effective. RBO Aleph is the first public web server implementing this novel *ab initio* prediction approach.

The effectiveness of our *ab initio* structure prediction algorithm has been demonstrated in 2014 during the most recent CASP experiment (http://www.predictioncenter.org/casp11). RBO Aleph was one of the top servers in the template-free modeling category.

To ensure the server's accuracy in the light of ongoing developments, we continuously monitor its performance through the CAMEO project (9).

Our web server provides an intuitive user interface, multiple visualization options and analysis tools to help users interpret the results. These include a visualization of the predicted model structures and contacts, the used modeling methods (template-based and/or *ab initio* modeling), used templates, detected domain boundaries and local prediction error estimates.

## MATERIALS AND METHODS

### RBO Aleph overview

**Figure 1.**
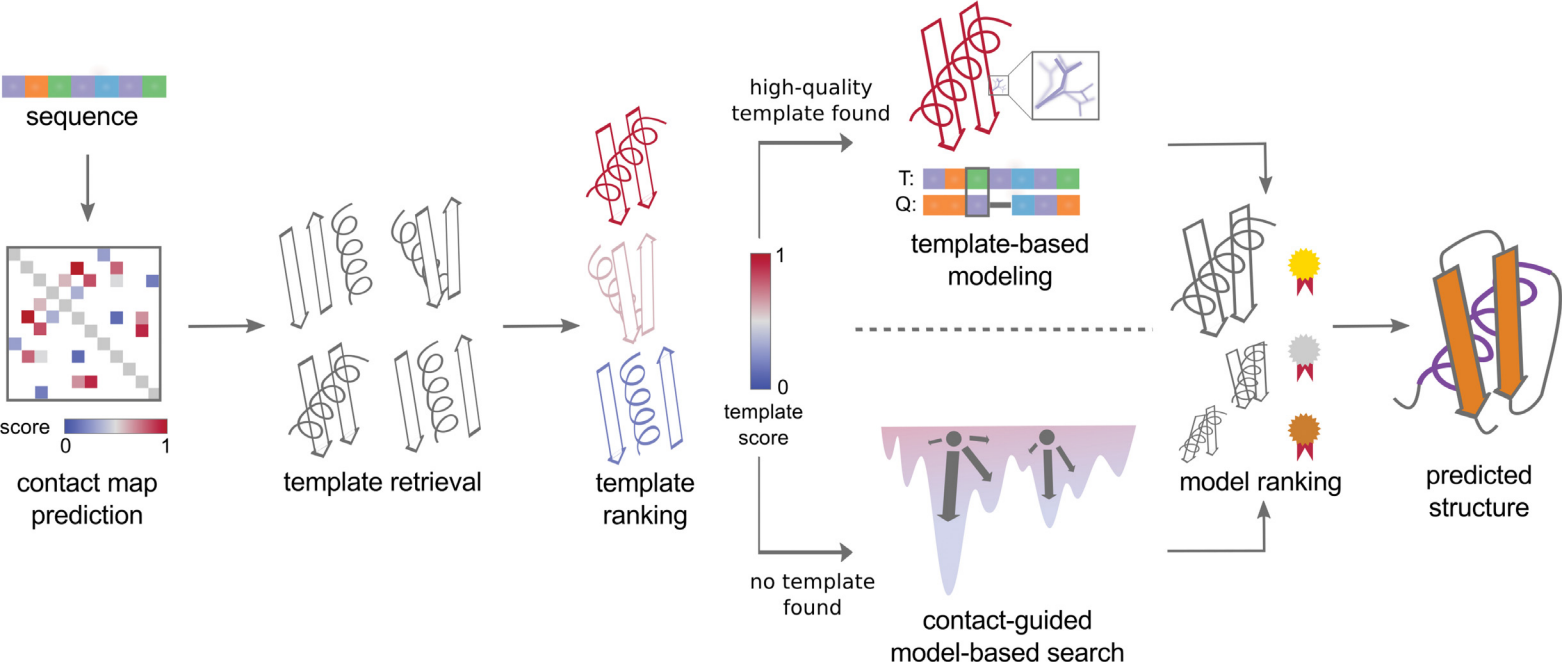
Overview of the RBO Aleph structure prediction pipeline: the server takes a protein sequence as input. First, residue–residue contacts are predicted using EPC-map (7). Then, template structures are identified with several threading algorithms (see ‘Materials and Methods’ section). A random-forest classifier re-ranks the templates. If suitable templates are identified, RBO Aleph performs template-based modeling with MODELLER (14). If no templates are available, RBO Aleph performs *ab initio* structure prediction with model-based search (MBS) (8) guided by contacts from EPC-map. After a final ranking by ProSA (16), the top five structures are selected as final predictions. For the sake of clarity, we omitted domain parsing and domain assembly steps for multi-domain proteins from this figure (see ‘Materials and Methods’ section for more detail on modeling of multi-domain proteins).

An overview of the RBO Aleph structure prediction method is given in Figure 1. Using a protein sequence as input, RBO Aleph performs contact prediction with EPC-map (7). Next, the server identifies candidate template structures with several threading algorithms: HHsearch (10), LOMETS (4), RaptorX (11) and SPARKS-X (5). The resulting templates are then re-ranked using a random forest classifier (12,13) trained based on scores from the different threading programs, consensus between template structures and compatibility between template structures and predicted contacts. The outcome of the classifier determines whether the structure will be modeled by template-based modeling or *ab initio* structure prediction.

If good templates are identified, our server performs homology modeling with MODELLER (14) and then uses a loop modeling protocol (15) to remodel parts of the protein not covered by the templates. In the absence of good templates, RBO Aleph performs *ab initio* structure prediction with model-based search (MBS) (8) guided by the predicted contacts. Next, RBO Aleph assesses the quality of the produced models using ProSA (16) and estimates the prediction error over the backbone using ModFOLD (17). Finally, the server returns the five highest-ranking predictions by ProSA's knowledge-based potential.

To accommodate multi-domain proteins, RBO Aleph attempts to split the query protein into domains. The server infers domain boundaries from retrieved templates. For long sequences without template, RBO Aleph uses a consensus score of two sequence-based domain prediction algorithms, PPRODO (18) and DomPro (19). If multiple domains are detected, the server predicts the structure of each domain individually and assembles them subsequently into a complete structure (20). Lastly, RBO Aleph assesses the quality of the predicted models using the Rosetta scoring function (21).

### Contact prediction in RBO Aleph

Residue–residue contact predictions from evolutionary sequence variation are accurate enough to improve three-dimensional structure prediction (22–24). However, these evolutionary algorithms perform poorly in the absence of deep alignments. EPC-map (7) leverages two complementary information sources to maintain high prediction accuracy in the absence of deep alignments. These two information sources are evolutionary and physicochemical information. Evolutionary information identifies residue–residue pairs that are close in space, which co-evolve to maintain structural stability. Physicochemical information identifies native contacts contained in predicted *ab initio* low-energy structures by evaluating the local physicochemical environment of a contact using a graph-based representation. This novel contact information plays a central role in our pipeline and is used in several parts of RBO Aleph (see ‘Leveraging evolutionary and physicochemical contacts in protein structure prediction’ section).

### *Ab initio* structure prediction

*Ab initio* structure prediction searches the conformational space of the protein for the native structure. The prediction success highly depends on the employed search strategy because the conformational space is large and the energy landscape is rugged. Model-based search (8) uses information from the exploration of the conformational space to build an approximate model of the energy landscape. This allows MBS to identify funnels in the energy landscape that most likely lead toward biologically relevant regions. Model-based search then allocates computational resources toward the most promising regions in the conformation space, thereby increasing the likelihood of sampling near-native structures.

### Leveraging evolutionary and physicochemical contacts in protein structure prediction

RBO Aleph leverages contact information from EPC-map at two stages in the modeling pipeline: to re-rank retrieved templates and to guide conformational space search.

RBO Aleph uses predicted contacts, in conjunction with other features, to evaluate and re-score templates with a random forest classifier (see ‘RBO Aleph overview’ section). The contact-based features characterize the ratio of satisfied contacts in each template, while taking into account the contact confidence estimated by EPC-map. High compatibility between predicted contacts and a template improves its ranking.

In addition, RBO Aleph uses contact restraints as energy terms to guide conformational space search. These additional energy terms smoothen the energy landscape, thereby effectively guiding search toward relevant regions.

Leveraging both evolutionary and physicochemical contact information in protein structure prediction is one of the key features of RBO Aleph.

### Method validation

We provide detailed evaluations of our contact prediction method (EPC-map) and of MBS in separate publications (7,8). We evaluated EPC-map in the most recent CASP11 experiment on 36 difficult protein domains (no detectable templates) in comparison to other state-of-the-art methods. EPC-map ranks first for long+medium-range contacts and seventh for long-range contacts (evaluation data from http://www.predictioncenter.org/casp11). The current version of RBO Aleph uses a revised set of parameters for EPC-map, further improving on the performance in CASP11.

We also evaluated RBO Aleph's tertiary structure modeling capabilities in CASP11. RBO Aleph ranked as one of the top servers in the template-free modeling category for 40 protein domains, demonstrating the effectiveness of MBS as a search strategy. RBO Aleph ranked first by the average *z*-score (>0) and third by the sum of *z*-scores (>−2) of the top prediction based on the assessors formula (evaluation data is taken from http://www.predictioncenter.org/casp11). Our server is competitive with established automated methods when considering all modeling categories. It ranked seventh by the average *z*-score (>0) and nineteenth by the sum of *z*-scores (>−2) of the top prediction.

In addition, RBO Aleph continuously participates in CAMEO (9) as a public server. CAMEO offers blind testing of modeling servers based on pre-released sequences whose structures will be published in the next PDB update. The current public version of RBO Aleph processed 99 predictions (from 21 November, 2014 to 13 February 2015) and its performance is compared in an ongoing fashion with other state-of-the-art structure prediction servers (data available at http://www.cameo3d.org/).

## RBO ALEPH SERVER

### Input

Users can submit prediction requests on the ‘Submit Job’ page. This requires a job name and the sequence of the query protein. The sequence has to be in the FASTA format, single-chained and shorter than 700 residues. Users may contact the authors to request predictions of larger proteins. Optionally, users can also provide residue–residue contact restraints. The server then linearly combines the input contacts with its own predicted contacts (equal weighting) and leverages the outcome as described in ‘Materials and Methods’ section.

Users can select a modeling protocol (template-based or *ab initio* modeling) to be exclusively used in prediction. They can also choose whether to activate the use of domains in modeling. By default, RBO Aleph automatically selects the modeling protocol and the domain boundaries.

After job submission, users receive an anonymized link to the future results page. Users can optionally provide an email address to receive the prediction results when they become available.

### Output

**Figure 2.**
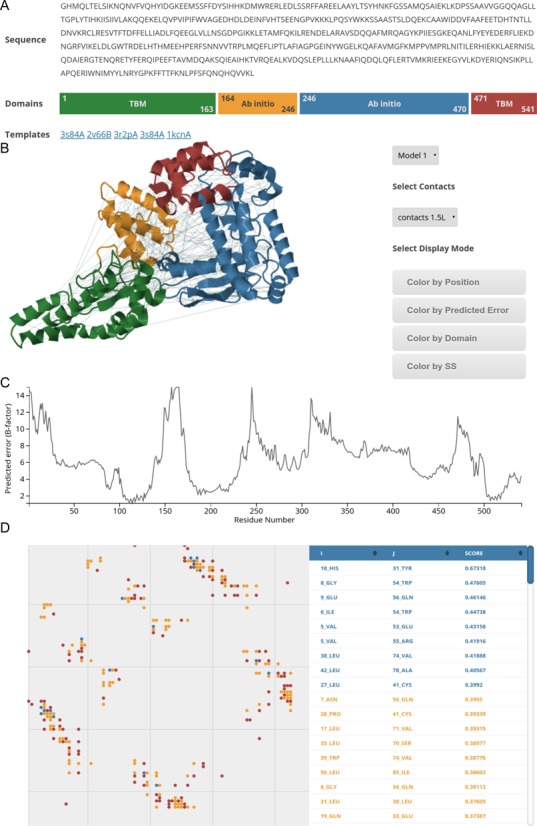
Example of prediction results: (**A**) General information regarding the modeling: color-coded depiction of predicted domains with residue boundaries and used modeling protocol (template-based modeling (TBM) or *ab initio* modeling) and list of templates used to model the protein. (**B**) Interactive 3D visualization of predicted contacts and structure: colors of the structural model refer to the domains in (A), the dotted lines represent the contacts. Drop-down menus provide easy access to inspect other models and subsets of contacts. The user can switch between different display modes, e.g. color the model based on the estimated prediction error or secondary structures (SS). (**C**) Local prediction error estimated over the whole model to distinguish poorly from well modeled parts. (**D**) Visualization of predicted contacts as interactive contact map and contact table. The table lists the residue numbers, amino acids and confidence scores for each contact. The number of depicted contacts automatically updates when the user chooses a different subset of contact in the 3D viewer (B).

RBO Aleph provides a number of tools to visualize the prediction results in the browser (Figure 2). The result page reports the modeling methods that were used and the predicted domains. If template-based modeling is used, our server presents a list of the used templates linked to their PDB pages.

The interactive 3-D viewer allows to easily inspect the predicted models. Users can choose to color models by residue position, secondary-structure, domain boundaries or prediction confidence. Furthermore, users can visualize the predicted contacts in the models. Per default, the viewer displays the top scoring *L*/10 contacts, where *L* refers to the sequence length. In addition, these contacts are visualized in an interactive contact map. The color of each contact denotes its prediction confidence. RBO Aleph uses 1.5*L* contacts to guide *ab initio* structure prediction.

RBO Aleph provides an estimate of the prediction error along the backbone in Ångström. This is plotted in the result page and updated whenever the user selects a new model.

The bottom of the results page offers download links for the prediction results. These include the predicted models in PDB format, the predicted contacts in CASP format and template alignments in FASTA format. The user can download the results individually or bundled as ZIP archive. RBO Aleph stores prediction results for two weeks, during which the results page will remain accessible. The user may contact the authors to extend the storage period.

### Processing time and server throughput

The required time for structure prediction highly depends on the protein difficulty, its length and domain count. Typically, the modeling of easy proteins takes 4–6 h. More difficult proteins requiring *ab initio* prediction need from 6 h up to 2 days. Practically, the duration of the prediction also depends on the number of jobs running in parallel on the server. On average, our server is able to process up to five simultaneous template-based modeling predictions and one *ab initio* prediction.

### Documentation

The documentation of RBO Aleph is accessible via the ‘Help’ menu on the web page. It includes details about the server, descriptions of input and output, explanations of prediction results and a section with frequently asked questions. Lastly, RBO Aleph presents the results of a sample prediction under the menu entry ‘Sample results’.

### Hardware and Software

RBO Aleph uses an IBM system cluster with 112 computing nodes. Each node is equipped with two quadcore CPUs with hyper-threading providing effectively 1792 slots for parallel computation. The web server's 3-D structure visualization builds on JSmol (25), while visualizations are rendered using a mixture of HTML5, Javascript and D3.js.

## CONCLUSION

We presented RBO Aleph, a novel server for fully automated protein structure prediction. The server performs template-based modeling, protein contact prediction and *ab initio* structure prediction.

RBO Aleph provides an intuitive user interface to our protein structure prediction pipeline. The server automatically selects the most promising modeling method, template-based modeling or *ab initio* structure prediction. The user can visually analyze the prediction results in the web browser and download the results for further analysis.

The main focus of RBO Aleph is *ab initio* prediction of difficult protein targets for which templates cannot be detected. The server uses MBS to effectively search the conformational space for native-like structures. In addition, we use residue–residue contacts derived from co-evolution and physicochemical information (EPC-map) to guide *ab initio* structure prediction. RBO Aleph is the first public available server that uses these methods.

RBO Aleph was among the best-performing methods in the template-free protein structure prediction category of CASP11. The performance of RBO Aleph is continuously monitored in CAMEO to ensure high prediction accuracy and reliability.
